# Low levels of neutralizing antibodies against XBB Omicron subvariants after BA.5 infection

**DOI:** 10.1038/s41392-023-01495-4

**Published:** 2023-06-19

**Authors:** Jingyun Yang, Weiqi Hong, Hong Lei, Cai He, Wenwen Lei, Yanan Zhou, Tingmei Zhao, Aqu Alu, Xuelei Ma, Jiong Li, Li Yang, Zhenling Wang, Wei Wang, Guangwen Lu, Guobo Shen, Shuaiyao Lu, Guizhen Wu, Huashan Shi, Xiawei Wei

**Affiliations:** 1 Laboratory of Aging Research and Cancer Drug Target, Department of Biotherapy and Cancer Center, National Clinical Research Center for Geriatrics, West China Hospital, Sichuan University, No. 17, Block 3, Southern Renmin Road, Chengdu, Sichuan 610041 People’s Republic of China; 2 NHC Key Laboratory of Biosafety, National Institute for Viral Disease Control and Prevention, Chinese Center for Disease Control and Prevention (China CDC), Beijing, 102206 China; 3 National Kunming High-level Biosafety Primate Research Center, Institute of Medical Biology, Chinese Academy of Medical Sciences and Peking Union Medical College, Kunming, Yunnan China

**Keywords:** Infection, Adaptive immunity

## Abstract

The COVID-19 response strategies in Chinese mainland were recently adjusted due to the reduced pathogenicity and enhanced infectivity of Omicron subvariants. In Chengdu, China, an infection wave was predominantly induced by the BA.5 subvariant. It is crucial to determine whether the hybrid anti-SARS-CoV-2 immunity following BA.5 infection, coupled with a variety of immune background, is sufficient to shape the immune responses against newly emerged Omicron subvariants, especially for XBB lineages. To investigate this, we collected serum and nasal swab samples from 108 participants who had been infected in this BA.5 infection wave, and evaluated the neutralization against pseudoviruses. Our results showed that convalescent sera from individuals, regardless of vaccination history, had remarkably compromised neutralization capacities against the newly emerged XBB and XBB.1.5 subvariants. Although post-vaccination with BA.5 breakthrough infection slightly elevated plasma neutralizing antibodies against a part of pseudoviruses, the neutralization activities were remarkably impaired by XBB lineages. Furthermore, we analyzed the impacts of the number of vaccinations, age, and sex on the humoral and cellular immune response after BA.5 infection. Our findings suggest that the neutralization against XBB lineages that elicited by current hybrid immunity after BA.5 infection, are remained at low levels, indicating an urgent need for the development of next-generation of COVID-19 vaccines that designed based on the XBB sub-lineages and other future variants.

## Introduction

**Fig. 1:**
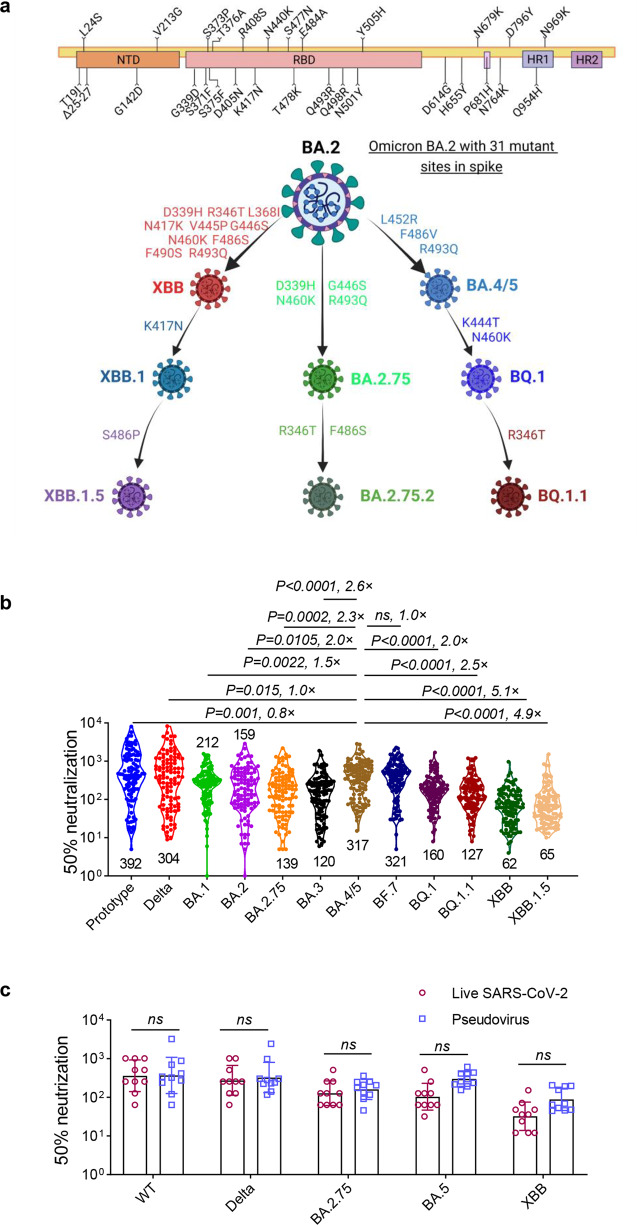
The neutralization against a variety of Omicron subvariants by convalescent sera from individuals recovered from BA.5 wave infection. **a** The schematic representation of the spike protein of SARS-CoV-2 Omicron BA.2 subvariants (up), and schematic depiction of the relationships between several circulating Omicron subvariants with the key amino acid substitutions (bottom). **b** The convalescent sera from 108 participants who infected with Omicron BA.5 subvariant from Dec 2022 to Jan 2023, in Chengdu, China were collected. Neutralizing antibody titers against prototype, Delta, BA.1, BA.2, BA.2.75, BA.3, BA.4/5, BF.7, BQ.1, BQ.1.1, XBB and XBB.1.5 pseudoviruses in convalescent sera were determined by pseudovirus neutralization assay. **c** Comparison of neutralizing antibody titers against live ancestral, Delta, BA.1, BA.2.75, BA.5 and XBB viruses. Data are presented as geometric mean values ± SD in (**b**, **c**). The GMT of 50% neutralization against pseudoviruses in (**b**) were only compared with BA.4/5 subvariant, and *P* values in (**b**) were determined by unpaired Student’s *t* tests, in (**c**) were performed by Two-way ANOVA followed by Sidak’s multiple comparisons test. ns not significant

Since its first emergence in South Africa in November 2021, the B.1.1.529 (Omicron) variant, with a large number of mutations in spike protein, has continued to circulate across the world while rapidly evolving into numerous descendant subvariants. The initial BA.1 was quickly supplanted by BA.2 and further evolved into a diverse array of subvariants including BA.2.75, BA.2.75.2, BA.4/5, BA.4.6 and BF.7.^1^ Following the dominance of BA.5, the new Omicron subvariant BQ.1 and BQ.1.1, which evolved from BA.5 (Fig. 1a), dramatically expanded in many countries.^2,3^ Recently, a new subvariant XBB lineage resulting from a recombination event between two BA.2 lineages (BA.2.10.1 and BA.2.75) has been first discovered in India.^3,4^ It has multiple mutations that are critical for the immune evasion functions, including R346T, G446S, and F486S.^4^ XBB.1.5, a descendant of XBB, with an additional substitution (S486P) (Fig. 1a), has been reported in several countries and become the predominant variant in the world.^5–7^ According to the U.S. Center for Disease Control and Prevention (CDC),^8^ as of April 1, 2023, XBB.1.5 accounts for 87.9% of currently circulating strains in the US, and a similar upward trend is expected to occur in numerous additional countries soon.

Although the pathogenicity of XBB lineages remain relatively low, their enhanced transmissibility and higher extent of immune escape raise grave concerns that these subvariants could substantially resist the neutralization induced by previous infection and vaccination efforts. Recent studies have reported that the extraordinary immune escape properties of XBB lineages, with the titers of neutralizing antibodies against these subvariants being significantly lower in individuals who have received the fourth mRNA boost shot or have Omicron BA.2 and BA.5 breakthrough infections.^1,3–6,9,10^ Bivalent vaccines that target the spike protein of ancestral wild-type (D614G) and BA.4/5 have been authorized for emergence use to confer protection against the new emerged Omicron subvariants. This bivalent booster exhibits a stronger ability to elicit higher neutralization responses against BA.5-derived subvariants than the parental vaccines.^1,11,12^ However, the bivalent vaccines could not produce robust neutralizing antibodies against the XBB lineages.^1,10,13,14^

The immune status of the population has become increasingly complex and heterogenous due to exposure to different vaccines, with or without infection by different SARS-CoV-2 variants, especially by Omicron and its subvariants.^15^ Omicron breakthrough infection may be considered as an adequate booster, significantly increasing the plasma neutralizing antibody titers in pre-immune people, rather than unvaccinated individuals.^16–21^ However, the immune response elicited by breakthrough infection depends on the previous vaccinations and SARS-CoV-2 exposure histories, as immune imprinting may occur.^22–25^ A recent study showed that the BA.5 breakthrough infection significantly reduces the epitope diversity of the neutralizing antibodies, suggesting the humoral immune repertoire elicited by BA.5 breakthrough may not be effectively diversified to neutralize future emerged subvariants.^26^ These studies strongly emphasize the need to investigate how breakthrough infections with different Omicron subvariants affect the neutralization against the further circulating variants such as XBB lineages.

Chinese “dynamic zero-COVID” policy has effectively blocked the spread of SARS-CoV-2 since March 2020. However, with the emergence of the Omicron variant and its subvariants, which have more rapid transmission and reduced pathogenicity, the Chinese government recently adjusted its COVID-19 response strategies.^27^ From December 2022 to January 2023, a huge wave of infections in China was predominantly caused by BA.5 and BF.7 subvariant. According to the Chinses and Sichuan Center for Disease Control and Prevention (CDC), the BA.5.2 subvariants was the dominant circulating variant during this infection wave in Chengdu, China. The most widely administered COVID-19 vaccines in mainland China are inactivated vaccines, including BBIBP (Beijing Institute of Biological Products Co., Ltd.), and CoronaVac (Sinovac Life Sciences Co., Ltd).^28^ Therefore, there is a need for a systematic evaluation of the neutralization abilities induced by BA.5 infection in the unvaccinated individuals or those vaccinated with inactivated vaccines, to confer protection against recently emerged Omicron subvariants.

For the present study, we recruited 108 participants from Chengdu, China, who were infected with the BA.5 variant between December 2022 and January 2023. These participants had varying vaccination histories with inactivated vaccines. The serum and nasal swabs samples were collected in the convalescent phase to determine binding and neutralizing antibodies responses. We also assessed the T cell response induced by previous infection and vaccination. This study aims to determine whether the immune response shaped by BA.5 infection, combined with the vaccination of inactivated vaccines, is sufficient to protect against the newly emerged XBB subvariants, and to emphasize the significance of developing of next generation of XBB lineage-specific COVID-19 vaccines.

## Results

### Low levels of neutralizing antibodies in convalescent sera from individuals who have been infected with BA.5 subvariant

To evaluate the neutralizing abilities of convalescent sera against pre-Omicron variants and Omicron subvariants, especially the rising XBB subvariants, we collected serum samples from 108 participants who had recently been infected and divided them into different clinical cohorts. Table 1 summarizes the demographic characteristics of the all participants, who ranged in age from 21 to 83 and had not received any vaccines or had received at least two doses of inactivated SARS-CoV-2 vaccines. Table 2 depicts the details of the vaccination histories of the participants. Among the adult participants (aged from 18 to 65), 39 had received three doses of inactive-virus vaccine before infection (adult 3 doses of IV group), 25 had received the two doses of vaccines before infection (adult 2 doses of IV group), and 22 were unvaccinated and infected with BA.5 (adult unvaccinated group). Besides the adult participants, there are 22 elderly participants (aged over 65 years) recovered from this BA.5 infection wave were rolled into this study, and all of them have received three doses of vaccines (elderly group). All the participants in this study provided written informed consent. Given the high prevalence of Omicron BA.5 infection from December 2022 to January 2023 in Chengdu, China, we hypothesized that the most participants were probably infected with BA.5 subvariants.

**Table 1:** Study cohort characteristics of COVID-19 patients in Chengdu, China, from December 2022 to January 2023

Characteristic	All patients (*n* = 108)	Vaccination status	
		Vaccinated (*n* = 86)	Unvaccinated (*n* = 22)
Age-yr	39 (21–83)	41 (21–83)	31 (24–41)
Gender			
Male	46 (42.6)	38 (44.2)	8 (36.4)
Female	62 (57.4)	48 (55.8)	14 (63.6)
Vaccination status in Adult^**#**^			
3 dose of IV	39 (45.3)	39 (60.9)	0 (0.0)
2 dose of IV	25 (29.1)	25 (39.1)	0 (0.0)
Unvaccinated	22 (25.6)	0 (0.0)	22 (100)
Vaccination status in Elderly*			
3 dose of IV	22 (100)	22 (100)	0 (0.0)

Adult^#^: participants ranged in age from 18 to 65; Elderly*: participants were over 65 years

**Table 2:** Details of vaccination histories of study participants recovered from SARS-CoV-2 BA.5 infection, Chengdu China, December 2022– January 2023 (*n* = 108)

Volunteer	Sex	Age	The first dose	The second dose	The third dose	Group
1	Male	34	/	/	/	Unvaccinated group (*n* = 22): 22 adult participants have not received injection of vaccine with infection
2	Female	30	/	/	/	
3	Female	30	/	/	/	
4	Female	25	/	/	/	
5	Female	32	/	/	/	
6	Male	30	/	/	/	
7	Female	31	/	/	/	
8	Female	28	/	/	/	
9	Female	31	/	/	/	
10	Female	33	/	/	/	
11	Female	32	/	/	/	
12	Male	29	/	/	/	
13	Male	24	/	/	/	
14	Female	27	/	/	/	
15	Male	24	/	/	/	
16	Female	31	/	/	/	
17	Male	31	/	/	/	
18	Male	33	/	/	/	
19	Female	35	/	/	/	
20	Female	37	/	/	/	
21	Male	35	/	/	/	
22	Female	41	/	/	/	
23	Male	27	CoronaVac	CoronaVac	/	2 doses of IV group (*n* = 25): adult participants who have received two doses of inactivated vaccines with infection
24	Female	28	CoronaVac	CoronaVac	/	
25	Male	25	CoronaVac	CoronaVac	/	
26	Female	24	CoronaVac	CoronaVac	/	
27	Male	31	CoronaVac	CoronaVac	/	
28	Male	21	BBIBP	BBIBP	/	
29	Female	25	CoronaVac	CoronaVac	/	
30	Female	25	BBIBP	BBIBP	/	
31	Male	25	CoronaVac	CoronaVac	/	
32	Female	25	BBIBP	BBIBP	/	
33	Female	31	CoronaVac	CoronaVac	/	
34	Male	24	CoronaVac	CoronaVac	/	
35	Male	27	CoronaVac	CoronaVac	/	
36	Female	24	CoronaVac	CoronaVac	/	
37	Male	31	CoronaVac	BBIBP	/	
38	Male	31	BBIBP	BBIBP	/	
39	Female	33	BBIBP	BBIBP	/	
40	Male	35	CoronaVac	BBIBP	/	
41	Female	37	CoronaVac	BBIBP	/	
42	Male	35	CoronaVac	CoronaVac	/	
43	Male	41	CoronaVac	CoronaVac	/	
44	Female	43	CoronaVac	CoronaVac	/	
45	Female	31	CoronaVac	CoronaVac	/	
46	Male	47	CoronaVac	CoronaVac	/	
47	Female	49	CoronaVac	CoronaVac	/	
48	Female	23	CoronaVac	CoronaVac	CoronaVac	3 doses of IV group (*n* = 39): adult participants who have received three doses of inactivated vaccines with infection
49	Female	22	CoronaVac	CoronaVac	CoronaVac	
50	Male	29	CoronaVac	CoronaVac	CoronaVac	
51	Female	23	CoronaVac	CoronaVac	CoronaVac	
52	Male	25	CoronaVac	CoronaVac	CoronaVac	
53	Male	29	BBIBP	BBIBP	BBIBP	
54	Female	52	CoronaVac	CoronaVac	CoronaVac	
55	Female	29	BBIBP	BBIBP	BBIBP	
56	Female	28	CoronaVac	CoronaVac	CoronaVac	
57	Female	25	CoronaVac	CoronaVac	CoronaVac	
58	Female	24	CoronaVac	CoronaVac	CoronaVac	
59	Female	24	CoronaVac	BBIBP	BBIBP	
60	Female	24	CoronaVac	BBIBP	BBIBP	
61	Female	23	CoronaVac	CoronaVac	CoronaVac	
62	Female	35	CoronaVac	CoronaVac	CoronaVac	
63	Female	30	CoronaVac	CoronaVac	BBIBP	
64	Female	23	CoronaVac	CoronaVac	CoronaVac	
65	Male	28	CoronaVac	BBIBP	BBIBP	
66	Female	31	CoronaVac	CoronaVac	CoronaVac	
67	Female	28	CoronaVac	CoronaVac	CoronaVac	
68	Female	41	CoronaVac	CoronaVac	CoronaVac	
69	Female	27	CoronaVac	CoronaVac	CoronaVac	
70	Male	35	CoronaVac	CoronaVac	CoronaVac	
71	Male	42	CoronaVac	CoronaVac	CoronaVac	
72	Female	25	CoronaVac	CoronaVac	CoronaVac	
73	Female	41	CoronaVac	CoronaVac	CoronaVac	
74	Female	27	CoronaVac	CoronaVac	CoronaVac	
75	Female	36	CoronaVac	CoronaVac	CoronaVac	
76	Male	39	BBIBP	BBIBP	BBIBP	
77	Male	28	CoronaVac	CoronaVac	CoronaVac	
78	Female	31	CoronaVac	CoronaVac	CoronaVac	
79	Male	28	CoronaVac	CoronaVac	CoronaVac	
80	Female	41	BBIBP	BBIBP	BBIBP	
81	Male	27	BBIBP	BBIBP	BBIBP	
82	Male	35	CoronaVac	BBIBP	BBIBP	
83	Female	42	CoronaVac	BBIBP	BBIBP	
84	Female	25	CoronaVac	BBIBP	BBIBP	
85	Male	41	BBIBP	BBIBP	BBIBP	
86	Female	27	CoronaVac	CoronaVac	CoronaVac	
87	Male	73	BBIBP	BBIBP	BBIBP	Elderly group (*n* = 22): elderly participants who were over 65 years with infection
88	Female	71	CoronaVac	CoronaVac	CoronaVac	
89	Male	66	CoronaVac	CoronaVac	CoronaVac	
90	Male	66	CoronaVac	CoronaVac	CoronaVac	
91	Male	69	CoronaVac	CoronaVac	CoronaVac	
92	Male	79	CoronaVac	CoronaVac	CoronaVac	
93	Male	83	CoronaVac	CoronaVac	CoronaVac	
94	Female	69	CoronaVac	CoronaVac	CoronaVac	
95	Female	73	CoronaVac	CoronaVac	CoronaVac	
96	Male	71	CoronaVac	CoronaVac	CoronaVac	
97	Male	72	CoronaVac	CoronaVac	CoronaVac	
98	Female	70	CoronaVac	CoronaVac	CoronaVac	
99	Male	73	CoronaVac	CoronaVac	CoronaVac	
100	Female	71	CoronaVac	CoronaVac	CoronaVac	
101	Male	66	CoronaVac	CoronaVac	CoronaVac	
102	Female	66	CoronaVac	CoronaVac	CoronaVac	
103	Male	69	CoronaVac	CoronaVac	CoronaVac	
104	Male	79	CoronaVac	CoronaVac	CoronaVac	
105	Female	83	CoronaVac	CoronaVac	CoronaVac	
106	Female	69	CoronaVac	CoronaVac	CoronaVac	
107	Male	73	CoronaVac	CoronaVac	CoronaVac	
108	Female	71	CoronaVac	CoronaVac	CoronaVac	

CoronaVac: inactivated vaccines from Sinovac Life Sciences Co., Ltd

BBIBP: inactivated vaccines from Beijing Institute of Biological Products Co., Ltd

We first assessed the neutralizing antibodies titers in all clinical cohorts against a series of pseudovirues in the panels (Fig. 1b). After infection, the geometric mean titers (GMTs) of 50% neutralization in convalescent sera to prototype and Delta were 392 and 304, respectively. The titers to Omicron subvariants, including BA.1, BA.2, BA.2.75, BA.3, BA.4/5, BF.7, BQ.1, and BQ.1.1 were 212, 159, 139, 120, 317, 321, 160 and 127, respectively. Consistent with previous studies reporting the extraordinary antibodies evasion properties of XBB lineages, the GMTs of 50% neutralization to the XBB and XBB.1.5 were only 62 and 65, and were remarkably lower than the titers to BA.5 by 5.1-, and 4.9-fold, respectively (Fig. 1b). These results indicated that recently emerged XBB subvariants extensively evaded the neutralizing antibodies in plasma of convalescent individuals, regardless of whether they are vaccinated or not, which suggests a high risk of reinfection.

We randomly selected several serum samples from all participants to perform an authentic virus neutralization assay (Fig. 1c). Encouragingly, the GMT of 50% neutralization were similar in both authentic virus and pseudovirus neutralization assays, with no statistical difference between the results obtained from the two systems. This indicates that our pseudovirus neutralization assay system used in this study can reliably reflect the results of live virus neutralization assay to some extent, and more accurately predict the neutralization capacities against XBB.1.5-included subvariants.

### Omicron XBB lineages remarkably compromised neutralization in vaccinated individuals with BA.5 breakthrough infection

**Fig. 2:**
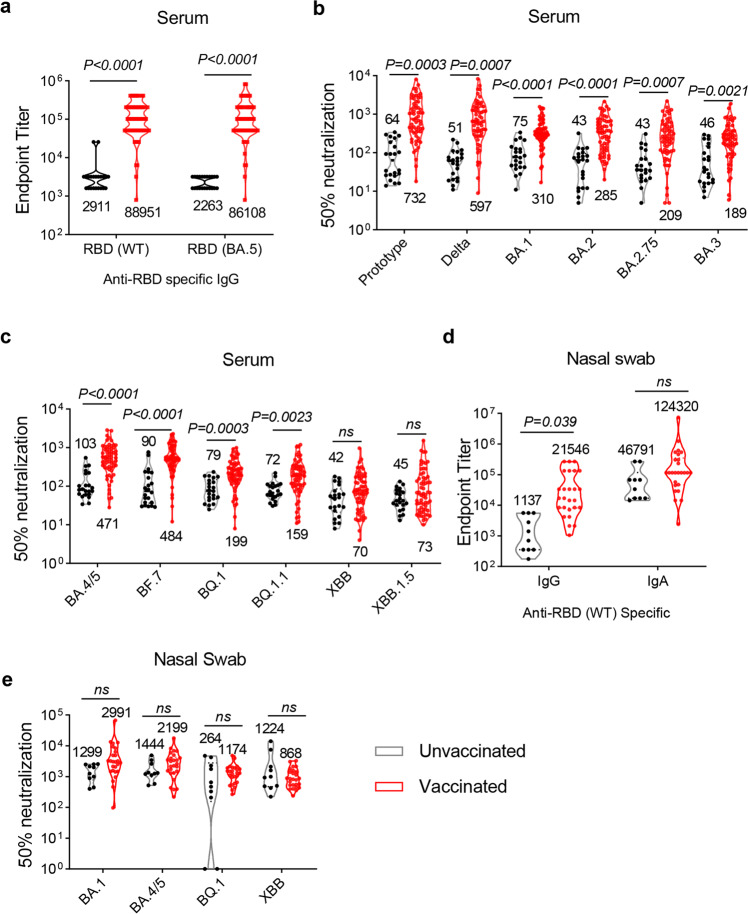
Comparison of humoral immune response in BA.5 infected-individuals paired with or without vaccination of inactivated vaccines. **a** The adult participants (aged from 18–65) were divided into two cohorts according to their vaccination status. There are 22 patients have not received injection of vaccine (unvaccinated group) with infection, and 64 have received at least two doses of inactivated vaccines (vaccinated group) with infection (25 received two doses and 39 received three doses of vaccines). The endpoint titers of wildtype and BA.5 spike-specific IgG antibodies in convalescent sera were determined by ELISA. **b** Neutralizing antibody titers against prototype, Delta, BA.1, BA.2, BA.2.75 and BA.3 pseudoviruses in convalescent sera from adult participants with or without vaccination. **c** Neutralizing antibody titers to BA.4/5, BF.7, BQ.1, BQ.1.1, XBB and XBB.1.5 pseudoviruses in convalescent sera. (*n* = 22 in unvaccinated group, and *n* = 64 in vaccinated group in **a**–**c**). **d** Endpoint titers of BA.5 spike-specific binding antibodies IgG and IgG in nasal samples from adult participants with different vaccination status. **e** Neutralizing antibody titers to BA.1, BA.4/5, BQ.1 and XBB pseudoviruses in nasal swab samples. (*n* = 10 in unvaccinated group, and *n* = 26 in vaccinated group in **d**, **e**). Data are presented as geometric mean values ± SD in (**a**–**e**). *P* values in (**a**–**e**) were determined by unpaired Student’s *t* tests. ns not significant

Previous studies reported the Omicron breakthrough infection improves cross-neutralization activities in pre-vaccination individuals.^16–21^ Thus, we wonder whether vaccination with inactivated vaccines could improve the neutralization capacities to protect against XBB.1.5-included Omicron subvariants. The adult participants (aged from 18 to 65) were divided into two clinical cohorts: individuals who had not received SARS-CoV-2 vaccine (unvaccinated, *n* = 22), and individuals who had received two (2 doses of IV, *n* = 25) or three doses (3 doses of IV, *n* = 39) of inactivated vaccines (vaccinated, total 64 participants). It is worth noting that serum samples from unvaccinated individuals had significant lower titers of binding antibody (Fig. 2a) and neutralizing antibody (Fig. 2b, c) after infection, with the GMTs of neutralization against the pseudoviruses ranged from 42 to 103. Compare with the unvaccinated individuals, the titers of neutralizing antibodies against a part of pseudoviruses were elevated in vaccinated group (Fig. 2b). However, the XBB lineages pseudoviruses remarkably impaired the neutralizing potency in vaccinated individuals’ sera, with the neutralizing antibody titers against XBB and XBB.1.5 were only 70 and 73, respectively (Fig. 2c). Vaccinated individuals after BA.5 breakthrough infection showed a 6.7-fold lower GMTs against XBB compared with the BA.5, and the decrease was 6.5-fold lower for XBB.1.5. These results suggested that the despite that improvements in neutralizing antibodies were coffered by the previous vaccinations, the neutralization capacities against the XBB lineages remained low levels after BA.5 infection in vaccinated individuals.

Measuring antibodies in respiratory tract is more informative for assessing the protection against SARS-CoV-2 infection because they can immediately neutralize the virus at the first line of entry.^15,29^ Therefore, we next assessed the titers of binding and neutralizing antibodies in nasal swab samples to determine the mucosal immune response (Fig. 2d, e). Although the endpoint titers of IgG in respiratory tract were slightly higher in vaccinated individuals, there was no significant improvement in antigen-specific IgA levels and neutralization capacities between two group. Specifically, the 50% neutralization GMTs to the BA.1, BA.4/5, BQ.1 and XBB subvariants in nasal swab samples from unvaccinated individuals were determined to be 1299, 1444, 264, and 1224, respectively, and titers in the vaccinated group were 2991, 2199, 1174 and 868, respectively, indicating that the pre-vaccination with inactivated vaccines did not strengthen the immune response in respiratory tract established by BA.5 infection.

### No significant difference in the post-infection improvement of neutralization abilities between two and three doses of immunizations

**Fig. 3:**
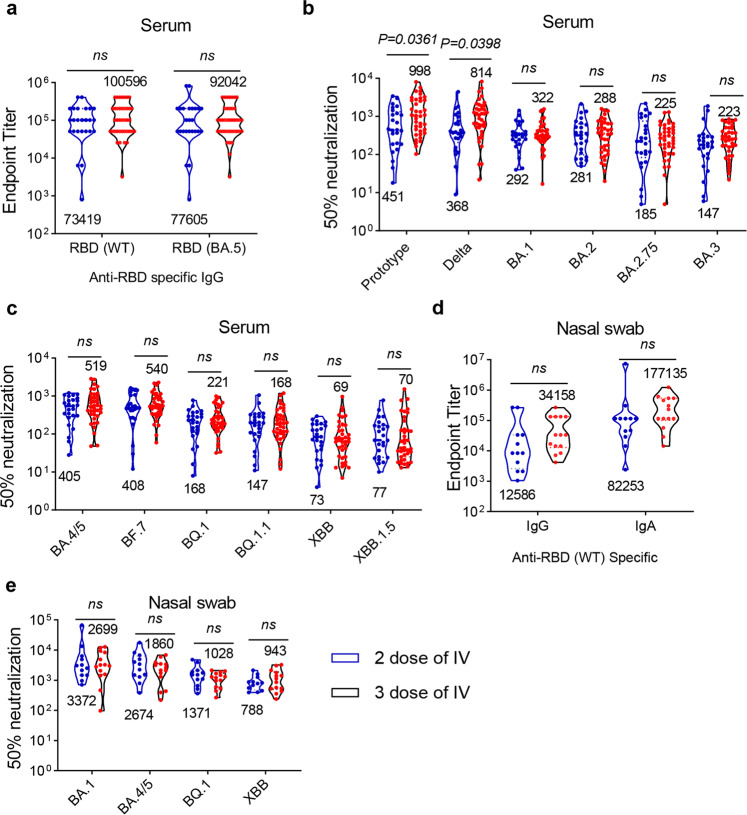
Comparison of humoral immune response after BA.5 infection with different number of vaccinations. **a** 64 adult (aged from 18–65) participants who have received injections of inactivated vaccines with infection were then divided into two cohorts: two doses of inactivated vaccine group (*n* = 25), and 3 doses of inactivated group (*n* = 39). The endpoint titers of wildtype and BA.5 spike-specific binding antibodies in sera were determined. **b** Neutralizing antibody titers against prototype, Delta, BA.1, BA.2, BA.2.75, BA.3 pseudoviruses in sera from adult participants with different number of vaccinations. **c** Neutralizing antibody titers to BA.4/5, BF.7, BQ.1, BQ.1.1, XBB and XBB.1.5 pseudoviruses in serum samples. (*n* = 25 in 2 dose of IV group, and *n* = 39 in 3 dose of IV group in **a**–**c**). **d** Endpoint titers of wildtype spike-specific binding antibodies IgG and IgG in nasal samples. **e** Neutralizing antibody titers to BA.1, BA.4/5, BQ.1 and XBB pseudoviruses in nasal swab samples. (*n* = 12 in 2 dose of IV group, and *n* = 14 in 3 dose of IV group in **d**, **e**). Data are presented as geometric mean values ± SD in (**a**–**e**). *P* values were determined by unpaired Student’s *t* tests. ns not significant

Several studies have reported the effects of different number of vaccinations on neutralization in BA.1 and BA.2 breakthrough infection.^16,18,19,30^ To investigate whether the number of vaccinations impacts the neutralizing potency after BA.5 infection, we compared the levels of binding and neutralizing antibodies in serum samples from infected adult individuals who have received two (2 doses of IV, *n* = 25) or three (3 doses of IV, *n* = 39) doses of inactivated virus vaccines (Fig. 3a–c). Although the titers of antigen-specific IgG (Fig. 3a) and neutralizing antibodies (Fig. 3b, c) in sera were slightly increased in the three doses of vaccine group, there was no statistically significant difference between the two groups except for the neutralizing antibodies against prototype and Delta variants. Of note, XBB lineages showed a greater degree of immune escape in the three-dose of vaccination group than in the two-dose group. The GMTs of 50% neutralization to XBB and XBB.1.5 subvariants in two-dose vaccination group were lower than BA.5 by 5.5- and 5.3-fold, respectively, and were lower by 7.5- and 7.4-fold, respectively, in three-dose group. Furthermore, the levels of IgG, IgA, and neutralizing antibodies in nasal swab samples were not significantly improved by a third-doses of boost shot (Fig. 3d, e). These results suggest that the improvement in neutralization capacities provided by vaccination after infection may be independent of the boosting status.

### Effects of age and sex on humoral immune response after BA.5 infection

**Fig. 4:**
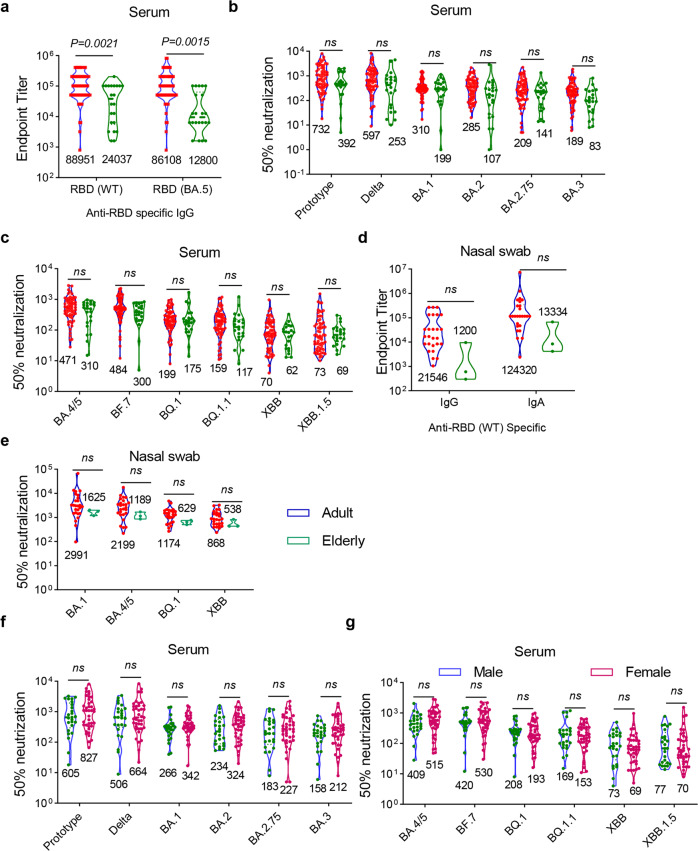
Impacts of age and sex on humoral immune response after BA.5 infection. **a** The endpoint titers of wildtype and BA.5 spike-specific binding antibodies in convalescent sera from 64 vaccinated adult (adult group) and 22 elderly (elderly group) participants. **b** Neutralizing antibody titers against prototype, Delta, BA.1, BA.2, BA.2.75, BA.3 pseudoviruses in convalescent sera in adult and elderly group. **c** Neutralizing antibody titers to BA.4/5, BF.7, BQ.1, BQ.1.1, XBB and XBB.1.5 pseudoviruses in serum samples. (*n* = 64 in vaccinated adult group, and *n* = 22 in vaccinated elderly group in **a**–**c**). **d** Endpoint titers of wildtype spike-specific binding antibodies IgG and IgG in nasal samples from vaccinated adult and elderly participants. **e** Neutralizing antibody titers to BA.1, BA.4/5, BQ.1 and XBB pseudoviruses in nasal swab samples from vaccinated adult and elderly participants. (*n* = 26 in vaccinated adult group, and *n* = 3 in vaccinated elderly group in **d**, **e**). **f** 64 vaccinated adult participants were divided into male (*n* = 25) and female group (*n* = 39) according their sex. The endpoint titers of wildtype and BA.5 spike-specific binding antibodies in convalescent sera were determined. Neutralizing antibody titers against prototype, Delta, BA.1, BA.2, BA.2.75 and BA.3 pseudoviruses in convalescent sera in male and female group. **g** Neutralizing antibody titers to BA.4/5, BF.7, BQ.1, BQ.1.1, XBB and XBB.1.5 pseudoviruses in serum samples in male and female group. (*n* = 25 in male group, and *n* = 39 in female group in **f**, **g**). Data are presented as geometric mean values ± SD in (**a**–**g**). *P* values were determined by unpaired Student’s *t* tests. ns not significant

In addition to the factor of immunization status, we also analyzed the effects of age and sex (Fig. 4) on post-infection humoral immune response. Sera antigen-specific IgG were significantly higher in adult participants (Fig. 4a), and the endpoint titers against RBD from wild-type and BA.5 variants were increased by 3.7- and 6.7-fold in the adult group than in the elderly, respectively. While neutralizing antibodies tend to decrease with age during the acute phase, they do not vary significantly during the convalescent phase.^28^ Consistent with this, our results showed the neutralizing antibodies against most of pseudoviruses in the panels were relatively lower in the elderly group but did not vary significantly (Fig. 4b, c). This is likely due to the fact that we assessed the humoral immune response during the convalescent phase rather than the acute phase. Moreover, the endpoint titers of IgG and IgA (Fig. 4d) and GMTs of neutralizing antibodies (Fig. 4e) in nasal swabs were higher in adult group, whereas there was no statistical difference between the two groups. Finally, the result of pseudovirus neutralization assay showed that sex is not a critical factor for the neutralizing antibody titers in sera after infection (Fig. 4f, g).

### Cellular immune response after BA.5 subvariant infection

**Fig. 5:**
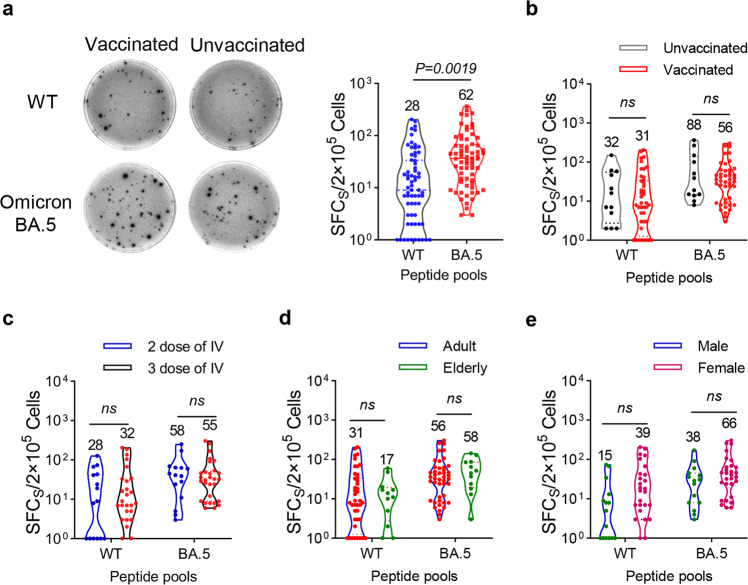
Cellular immune response after infection with BA.5 subvariant. **a** The lymphocytes in peripheral blood were collected, and the number of IFN-γ-secreting T cells were determined by ELISpot after stimulation with peptides pools of wildtype or BA.5 spike proteins. **b**–**e** Analysis of effects of on vaccination histories (**b**), number of vaccinations (**c**), age (**d**) and sex (**e**) on the number of IFN-γ-secreting T cells. Data are presented as mean with ± SEM in **a**–**e**. *P* values were determined by unpaired Student’s *t* tests. ns not significant

Apart from antibody response, T cell immune response is imperative for limiting the viruses, and is associated with protection against SARS-CoV-2 infection.^15,31^ To investigate the cellular e response induced by the BA.5 subvariant, we isolated and collected the peripheral blood mononuclear cells (PBMCs) from participants. The PBMCs were stimulated with overlapping peptide pools spanning the spike protein of ancestral and BA.5 strains. Both ancestral and Omicron spike induced cytokine secretion, while the T cellular immune response was stronger upon stimulation with BA.5 spike proteins, manifested by a higher frequency of IFN-γ-secreting cells (Fig. 5a). Contrary to our expectation, pre-vaccination did not strengthen cellular immune response after BA.5 infection as much as humoral immunity did (Fig. 5b). This result indicated BA.5 infection elicited a strong BA.5 variant-specific cellular immune response in both vaccinated and unvaccinated individuals. Furthermore, we found that the degree of specific T cell response was not significantly associated with the vaccination history (Fig. 5c), age (Fig. 5d) and sex (Fig. 5e).

## Discussion

Since its first detection in the United States in October 2022, the proportion of SARS-CoV-2 infections caused by the XBB lineage has steadily increased, making it the dominant SARS-CoV-2 lineage in the United States. A similar trend is expected to occur soon in European countries.^32^ From December 2022 to January 2023, China mainland experienced a COVID-19 wave caused by dominant variants BA.5 and BF.7, following the lifting of many strict ‘zero-COVID-19’ measures. It remains unclear whether the hybrid anti-SARS-CoV-2 immunity following BA.5 infection, paired with a variety of immune background, is enough to shape the immune responses against the next possible arrival of Omicron subvariants, such as XBB.1.5 subvariant. In the current study, we systematically compared the antibody and cellular immune responses of BA.5-infected individuals with or without different histories of inactivated vaccine vaccinations. Our results indicated BA.5 breakthrough infection further improves the immunity established by pre-vaccination with inactivated vaccines. However, the neutralization in convalescent sera with low potency against XBB lineages might not be able to protect against re-infection, underscoring the importance of development of next generation of SARS-CoV-2 vaccines that designed based on the sequence of XBB subvariants.

A large number of studies have demonstrated that pre-vaccination before infection elicits a stronger humoral immune response with broader spectrum neutralizing antibodies than unvaccinated individuals.^16–19^ In our study, we reported improvements in plasm neutralizing antibodies after BA.5 infection in patients who have received at least two doses of inactivated virus vaccines compared to those unvaccinated patients. Nevertheless, it is worth noting that the antigenic diversities between a variety of Omicron subvariants further complicate the immune response after infection.^33^ To be specific, although the pre-vaccination can improve the neutralization capacities after the Omicron breakthrough infection, the new emerged Omicron subvariants can remarkably escaped from the high neutralization potency induced by infection with previous subvariants. For example, neutralizing antibodies induced by BA.1 breakthrough are less efficient against BA.4/5 subvariants,^23,34^ and BQ and XBB lineages were less well recognized by convalescent sera from individuals who have received inactivated and mRNA vaccines in BA.1, BA.2, BA.5 or BF.7 breakthrough infection.^2,3,7,26,35^ In current study, we also demonstrated that the XBB and XBB.1.5 subvariants with extraordinary evasion abilities significantly compromised the neutralization induced by BA.5 infection, even in people who have previously received at least two doses of the inactivated virus vaccines. We reported the GMT of 50% neutralization against recently emerged XBB.1.5 in convalescent sera from unvaccinated and vaccinated groups were only 45 and 73, respectively. These findings indicate neutralization activities to XBB lineages after BA.5 infection are still weak to protect against infection.

In addition to immune escape properties due to antigenic diversity caused by mutations on spike proteins, neutralizing antibody responses to new Omicron subvariants might be hindered by prior antigenic exposure as the existence of immune imprinting.^36^ A previous study demonstrated repeated vaccination with inactivated vaccines dampens the immune responses to new Omicron subvariant in BA.2 breakthrough infection,^30^ highlighting the existence of immune imprinting. In addition, the immune imprinting was also observed in the post-vaccination infection with BA.5 subvariants, such that the breakthrough infection recalled cross-reactive memory B cells induced by vaccines based on original strain, whereas it hardly produced BA.5-specific B cells.^26^ These studies may explain why vaccinated people are breakthrough infected with the new Omicron subvariants, and suggests the next generation variant-specific vaccines should be urgently developed.

Several studies have reported the effect of number of vaccinations on neutralizing antibody levels in breakthrough infection.^16,18,19,30^ Although three-dose vaccination with inactivated vaccines exhibited better neutralizing capacity than two-dose vaccination in uninfected people,^37^ the number of vaccinations did not affect the neutralizing antibody titers after BA.1^16^ or BA.2^18^ infection. Consistent with these studies, we found no significant increase in neutralizing antibody titers after BA.5 infection in the three-dose vaccine group compared with the two-dose group. However, it has been reported third-dose of vaccination with mRNA vaccines enhanced cross-neutralization activities against a broad range of SARS-CoV-2 variants after Omicron breakthrough infection.^19^ Moreover, a recent study demonstrated three-dose of inactivated vaccine vaccination dampens the immune responses to new Omicron subvariants compared with two-dose vaccination in BA.2 breakthrough infection.^30^ These differences may be resulted from the different kind of vaccines and infection with different variants. Therefore, future studies may need to detailly investigate the effects of number of vaccinations with different vaccines on neutralizing antibody levels after different variants breakthrough infection.

In the present study, we also analyzed the impacts of the age and sex on the production of neutralization antibodies, and our results showed these conditions did not significantly affect the levels of neutralizing antibodies in sera. It has already been reported that the neutralizing antibodies decreased with the age in the acute phase (first two weeks after the disease onset), but not in convalescent phase (after two weeks of the disease onset). This may be because patient with different ages during this phase have sufficient time to yield the neutralizing antibodies.^28^ Indeed, the serum and nasal swab samples from participants in our studies were collected 2–4 weeks after recovery from the infection, representing considerable levels of neutralizing antibodies in convalescent phase. Previous study reported the circulating antibody levels in male were initial higher but more marked decrease.^38^ However, our study only focused on the effects of sex factor on neutralizing antibodies in sera collected at 2–4 weeks after infection, the more information about the dynamics of humoral immunity should be investigated in future studies.

The current study is limited by the small sample size, with only 108 participants enrolled. In addition, we did not test for the sequence of infection subvariant, and hypothesized that all participants in the current study were infected with BA.5 given that the dominant infection strain was BA.5.2 subvariant from December 2022 to January 2023 in Chengdu. In the current study, we solely utilized ELISpot assay to detect cytokine secretion of PBMCs after stimulation with spike protein pools to evaluate the T cell immune response. Incorporating other methods, such as flow cytometry, to detect the activation of various T cell subsets may provide a more accurate evaluation of cellular immune response. Nevertheless, our study systematically evaluated the neutralization activities of XBB.1.5-included Omicron subvariants following BA.5 infection, paired with different inactivated vaccines vaccination histories. This provides new insights into development of future vaccine matching newly emerged circulating variants.

## Methods

### Sample collection after infection

We collected blood samples and nasal swabs from volunteers in Chengdu, China, who were infected with the SARS-CoV-2 mutant BA.5 beginning in December 2022. Prior to enrollment, we requested that volunteers provide evidence of a previous SARS-CoV-2 antigen test confirming that they had been infected during the BA.5 infection wave. The samples were collected and stored at −80 °C until used. This research involving human participants was reviewed and approved by Ethics Committee Institution. We obtained written informed consent from all volunteers in the study.

### Materials

The RBD protein was produced by our group as previously reported,^39^ and the S-Omicron protein (CAT: SPN-C522e) was provided by ACROBiosystems. HRP-conjugated goat anti-human IgG (CAT: 62–8420) was purchased from Invitrogen, and IgA (CAT: 2050-05) antibody was purchased from SouthernBiotech. Pseudoviruses expressing luciferase, including Prototype, Delta and Omicron sublineages (BA.1, BA.2, BA.2.75, BA.3, BA.5, BF.7, BQ.1, BQ.1.1, XBB and XBB.1.5) were provided by Genomeditech.

### Enzyme-linked immunosorbent assays (ELISAs) for antibody measurement

We measured antigen-specific antibodies as previously described.^39^ Briefly, RBD or S-Omicron protein was coated onto 96-well ELISA plates (Thermo Scientific, United States) at a concentration of 1 μg/ml in sodium carbonate solution (50 mM, pH 9.6) and incubated at 4 °C overnight or at 37 °C for two hours. The plates were then washed three times with wash buffer (0.5% PBST: PBS containing 0.5% tween 20) and blocked with 1% BSA for 1 h at 37 °C. After washing the plates once, we added 100 μl dilutions of serum and nasal swabs to each well and incubated the plates at 37 °C for 1 h. The plates were then washed three times and 100 μl of anti-human IgG (1:20000) or anti-human IgA (1:4000) in 1% BSA was added to each well. After incubating at 37 °C for 1 h and washing the plates three times with PBST, we added 100 μl of 3,3′,5,5′-tetramethyl biphenyl diamine (TMB) and developed the plates for about 10 min at room temperature. Finally, we added 100 μl of 1.0 M H_2_SO_4_ to each well and measured the absorbance values at 450 nm on a microplate reader (Spectramax ABS, Molecular Devices). We defined endpoint titers as the lowest dilution at which the optical density (O.D.) was above 0.105.

### Pseudovirus neutralization assay

A pseudovirus neutralization assay was conducted following previously described methods.^40^ In brief, serum and nasal swabs samples were diluted by 1:3 dilution series in 96-well plates, resulting in a final volume of 100 μl per well. The stock solutions of pseudoviruses were diluted by culture medium, and the plates were then added to 50 μl of pseudovirus dilution (Prototype, Delta, BA.1, BA.2, BA.2.75, BA.3, BA.5, BF.7, BQ.1, BQ.1.1, XBB or XBB.1.5) and incubated in a 37 °C incubator for approximately 1 h. Subsequently, human ACE2 receptor expressing HEK-293T (293 T/ACE2) cells (100 μl 1.2 × 10^4^/well) were added to the plates containing the serum-virus mixture, and the plates were incubated for 48 h at a 37 °C incubator. After removing the supernatant, 100 μl of lysis reagent (Promega, USA) was added to the plates, and the luminescence was read using a multi-mode microplate reader (PerkinElmer, USA).

The positive control group contained only cells and viruses, the negative control group contained only cells, and the sample group contained cells, serum or nasal swab samples and viruses. The percentage of neutralization was calculated using the following equation:

$${\rm{neutralization}}\,\left( \% \right)=\left(\frac{{{{\rm{luminescence}}}_{{postive}}-{\rm{luminescence}}}_{{sample}}}{{{{\rm{luminescence}}}_{{postive}}-{\rm{luminescence}}}_{{negative}}}\right)\times 100 \%$$

### Live SARS-CoV-2 virus neutralization assay

To determine the neutralizing antibodies in sera convalescent against live SARS-CoV-2 viruses, we performed the authentic virus neutralization assay. Diluted serum samples were mixed with live SARS-CoV-2 viruses at 50% tissue-culture infectious doses (TCID50), followed by incubation at 37 °C for 1 h. The mixture was then added to 96-well microplates covered with Vero E6 cells (5 × 10^4^ /well) and incubated for 72 h. The cytopathogenic effects (CPE) were observed using a microscope, and the titers of neutralizing antibodies in the immunized sera resulting in EC50 (50% neutralization) inhibition were calculated.

### Enzyme linked immunospot assay (ELISpot) for cellular immune response assay

We first isolated peripheral blood mononuclear cells (PBMCs) by density gradient centrifugation using lymphocyte isolation sterile solution (Lot: 10321567, Cytiva), PBMCs were collected, washed 2 times with RPMI 1640 complete medium containing 10% serum, resuspended with cryopreservation solution and stored in a −80 °C freezer for later use.

96-well IFN-γ ELISpot plates (cat: 3420-4APT-2, MABTECH) were cleaned 4 times with PBS, added to 1640 complete medium (100 μl/well) and incubated at room temperature for 1 h. After removing the medium, 100 μl of PBMCs (2 × 10^5^/well) were added to the wells and stimulated overnight with WT or Omicron (BA.5) peptide pools in a 37 °C incubator. Then we removed the cells, washed the plates 5 times with PBS and added 100 μl detection antibody (7-B6-1-biotin,1 μg/ml) to each well and the plate were incubated for 2 hours at room temperature. Washing 5 times with PBS, the plates were added with Streptavidin-ALP (1:1000) and incubated for 1 h at room temperature. The plates were washed 5 times and developed using ready-to-us substrate solution (BCIP/NBT-plus). Once a noticeable spot emerged in the plate, we used clean water rinsing to stop the develop. At last, IFN-γ ELISpot plates were scanned on an AID ELISpot Reader.

### Statistical analysis

Statistical analyses were performed using Prism 9.0 (GraphPad software). The data are presented as geometric mean values ± SD. or mean with ±SEM, as indicated in each figure caption. The *P* values were determined using unpaired Student’s t-tests or Two-way ANOVA followed by Sidak’s multiple comparisons test as indicated in each figure legend. *P* values < 0.05 were considered significant, ns not significant.

## Data Availability

All data are available in the main text or the Supplementary Materials.
